# Identification and Role of Regulatory Non-Coding RNAs in *Listeria monocytogenes*

**DOI:** 10.3390/ijms12085070

**Published:** 2011-08-10

**Authors:** Benjamin Izar, Mobarak Abu Mraheil, Torsten Hain

**Affiliations:** 1 Institute of Medical Microbiology, Justus-Liebig-University, Frankfurter Strasse 107, Giessen 35392, Germany; E-Mails: Benjamin.Izar@med.uni-giessen.de (B.I.); Mobarak.Mraheil@mikrobio.med.uni-giessen.de (M.A.M.); 2 Department of Internal Medicine, Massachusetts General Hospital, 55 Fruit Street, Boston, MA 02118, USA

**Keywords:** *Listeria monocytogenes*, sRNA, asRNA, riboswitch, non-coding RNA, regulatory RNA, RNA-Seq, whole genome tiling arrays, infection, UTR

## Abstract

Bacterial regulatory non-coding RNAs control numerous mRNA targets that direct a plethora of biological processes, such as the adaption to environmental changes, growth and virulence. Recently developed high-throughput techniques, such as genomic tiling arrays and RNA-Seq have allowed investigating prokaryotic *cis*- and *trans*-acting regulatory RNAs, including sRNAs, asRNAs, untranslated regions (UTR) and riboswitches. As a result, we obtained a more comprehensive view on the complexity and plasticity of the prokaryotic genome biology. *Listeria monocytogenes* was utilized as a model system for intracellular pathogenic bacteria in several studies, which revealed the presence of about 180 regulatory RNAs in the listerial genome. A regulatory role of non-coding RNAs in survival, virulence and adaptation mechanisms of *L. monocytogenes* was confirmed in subsequent experiments, thus, providing insight into a multifaceted modulatory function of RNA/mRNA interference. In this review, we discuss the identification of regulatory RNAs by high-throughput techniques and in their functional role in *L. monocytogenes*.

## 1. Introduction

*Listeria monocytogenes* is a Gram-positive facultative intracellular bacterium that occurs ubiquitously in nature. Its genome bears a low G + C DNA content and is closely related to the genus of *Streptococcus*, *Staphylococcus*, *Enterococcus* and *Clostridium*. Eight different species have been described for the *Listeria* genus, including *Listeria monocytogenes*, *Listeria ivanovii*, *Listeria innocua*, *Listeria welshimeri*, *Listeria seeligeri*, *Listeria grayi*, *Listeria marthii* and the recently isolated *Listeria rocourtiae* [1–4].

*L. monocytogenes* cause infections in humans and animals, such as meningitis and septicaemia that are associated with a high mortality rate up to 30%, despite antibiotic therapy [5]. A characteristic feature in the pathogenesis of *L. monocytogenes* is the ability to actively induce its own phagocytosis by host cells mediated by internalins (*inl*) and escape from the phagosom/autophagosom using the virulence factors listerolysin (LLO) or ActA for actin polymerization. Once located in the host cytosol, it replicates and induces actin-based motion allowing the pathogen to move in infected cells and spread to adjacent cells [3]. With exception of *inlA-C* and *hpt* [6], the major virulence genes, responsible for the intracellular life cycle of *L. monocytogenes* are clustered in a ∼9 kb chromosomal region that is controlled by the principal virulence regulator PrfA [2,3,7].

*L. monocytogenes* has served as a model pathogen for the investigation of host-pathogen interactions and the immune response following infection intracellular bacteria and is now one of the best characterized microorganisms worldwide. Using recently developed high-throughput techniques, such as tiling arrays and next-generation DNA sequencing, which allows high-throughput RNA analysis through cDNA sequencing (RNA-Seq), several studies uncovered numerous non-coding RNAs in the listerial genome that were expressed under different conditions and introduce a new chapter of *L. monocytogenes* as an indispensable model organism for the investigation of prokaryotic transcriptomics [8–10].

Three major studies detected 180 regulatory RNAs that are almost exclusively non-coding [8–10]. Discovery of sRNAs in several other bacteria, including *Escherichia coli* [11,12], *Salmonella typhimurium* [13,14], *Pseudomonas aeruginosa* [15], *Streptococcus pyogenes* [16–18], *Streptococcus pneumoniae* [19] and *Staphylococcus aureus* [20] extended our view to an unexpected complexity of prokaryotic genomes and transcriptomic regulation. These studies also emphasized that the portion of non-coding RNAs was much higher than previously expected [21].

Non-coding RNAs control the fate of mRNA through different mechanisms and thereby influence several biological processes and virulence in *L. monocytogenes*. In general, interference of regulatory RNA with mRNA leads to destabilization and degradation of the target mRNA [22]. Interaction generally occurs through (i) *trans*-acting sRNAs encoded in intergenic regions (IGRs) at a chromosomal locus distal to the target transcript, (ii) anti-sense RNA (asRNA) encoded on the opposite strand of the open reading frame (ORF) or overlapping 5′ or 3′ untranslated region (UTR), and (iii) *cis*-regulatory RNAs, including riboswitches and 5′UTRs located upstream of a target gene with possible overlap with the ORF [23]. However, bifunctional regulatory RNAs, such as the SAM riboswitch that also acts as *trans*-acting sRNA (discussed below), have recently been described and highlight an unexpected level of complexity of prokaryotic transcriptional regulation [24].

In this paper, we give an overview on non-coding RNAs in *L. monocytogenes*, their role in virulence and adaptation to environmental changes predominantly identified by genomewide approaches.

## 2. Identification of Regulatory RNAs in *L. monocytogenes* Using High-Throughput Techniques

Gene expression analyses using microarrays provided us with comprehensive information on how *L. monocytogenes* adjusts to physiological and hostile environmental changes. For instance, previous studies investigated the intracellular expression signature and found that *Listeria* induces several metabolic and virulence genes that in concert allow survival and proliferation within host cells [25,26]. Further studies using mutant bacteria lacking transcription factor SigmaB (σ^B^) characterized several stress response genes that are important for intracellular survival of the pathogen [27,28]. However, due to the microarray chip design and technology, only intensities of transcripts from protein-coding regions are assessed and leave non-coding regions unexamined.

Two innovative techniques, genomic tiling arrays and RNA-Seq have revolutionized the field of RNA research, because they provide an uninterrupted picture of the whole transcriptome, including non-coding RNAs [9,10,13,29,30]. In both cases, isolated RNA is reverse transcribed to cDNA. Prior to reverse transcription in RNA-Seq, mRNA enrichment may be performed by reducing rRNAs and tRNAs levels from the total RNA sample [21]. In RNA-Seq, cDNA is then subjected to high-throughput sequencing. Resulting sequences are mapped to the genome and deliver a detailed snapshot of the current transcriptome. Whole genomic tiling array are microarray that cover the entire genome, regardless of the position of annotated ORFs, by overlapping oligonucleotide probes and permits the identification of non-coding regions. cDNA is hybridized to the array to measure intensity of each transcript which then allows to analyze expression profiles.

The first sRNA in *L. monocytogenes* has been identified by Barry *et al.* in 1999 [31]. Subsequently, additional 20 sRNAs were identified using northern blots and co-immunoprecipitation using Hfq, a protein that binds to sRNAs and stabilizes the hybridization to target mRNAs [32–34].

Using whole genome tiling arrays, Toledo-Arana *et al.* provided the first genome-wide study on the identification of regulatory RNA candidates expressed in *L. monocytogenes* and its deletion mutants under various physiological growth conditions in human blood, the intestinal lumen and other organs in mice [9]. They identified 103 putative regulatory RNA elements, several of which are absent in the non-pathogenic *L. innocua.* These data was largely confirmed by a following study using RNA-Seq by Oliver *et al.* who found 67 non-coding RNAs, of which 7 have not been described previously [8]. One *trans*-acting sRNA, *sbrE* was found to be directly regulated by σ^B^. However, due to the RNA-Seq protocol, regulatory RNAs previously described to be coded anti-sense were not covered. In a more recent study, however, Hain and colleges provided the so far most detailed picture on sRNAs in *L. monocytogenes* using RNA-Seq that also allowed for directional reads [10]. 150 small non-coding RNA elements were found, which enclosed 75% [9] and 69% [8], respectively, of previously reported regulatory RNAs. Differences in the number of regulatory RNAs was mainly due to 25 newly discovered asRNAs by Mraheil *et al.* [10]. Furthermore, comparison with a computational analysis for the prediction of regulatory RNAs in *L. monocytogenes* revealed that only 33% of predicted sRNAs overlapped with the RNA-Seq data [10,35].

## 3. How Non-Coding Regulatory RNAs Control the Fate of mRNA in *L. monocytogenes*

*L. monocytogenes* survives at a broad range of temperatures (0–45 °C) and pH (4.5–9), at high salt concentrations (10% NaCl) and also in most hostile environment, such as the phagocytic vacuole and within infected macrophages [2,3,10]. For this reason, sensing the surrounding and rapid adaptation to environmental changes is essential for the bacterium in order to survive in its ecological niches. Both tasks may be accomplished by non-coding regulatory RNAs by interfering with different structures of the target transcript or due to regulation at the transcriptional level. Compared to regulatory proteins, RNAs offer some advantages, because they present a rapid connection between quorum sensing and direct destabilization of target mRNA, and allow a fine tuning of the response at a post-transcriptional level. In addition, unnecessary regulatory RNAs may be cleared quickly using less energy. Therefore, it is not surprising that several bacteria maintain a considerable set of regulatory RNAs that account for at least 3–13% of the whole genome [21].

## 4. 5′UTRs and Multi-Functional Regulatory RNAs

5′UTRs are defined as the mRNA region starting at the promoter and ends with the start codon of the ORF. Since the transcription of an ORF may begin at different promoters, the length of the 5′UTR of a protein coding transcript may vary in length. 5′UTRs act as sensors for temperature and metabolites in *L. monocytogenes* and affect virulence and growth at the same time.

PrfA*,* the master virulence regulator in *L. monocytogenes*, is controlled by a thermosensor that is located 116 nucleotides 5′UTR upstream of the coding mRNA sequence [36]. At low temperatures (30 °C), the 5′UTR forms a complex secondary structure that hides the Shine–Dalgarno (SD) region and thus prevents the translation of *prfA.* An increase in temperature to 37 °C leads to formation of an alternative secondary structure that uncovers the SD site and allows for translation of the *prfA* transcript. Subsequently, PrfA induces expression of e.g. the major virulence factors LLO and ActA.

Metabolite-binding 5′UTRs that act as sensors for a substrate are also known as riboswitches. These RNA control elements possess an aptamer, an RNA region that is specific to a metabolite (e.g., lysine). Binding of the metabolite leads to an alteration of the riboswitch structure and results in transcription termination of the downstream gene. Several putative riboswitches have been identified for *L. monocytogenes* [9,10,24,37], of which the lysine riboswitch (LysRS) is one of the best characterized. The LysRS is located 280 nucleotides 5′UTR upstream of a lysine transporter gene (*lmo0798*). When present in abundance, lysine binds to the LysRS and stabilizes its tertiary structure leading to the formation of a Rho-independent transcriptional terminator (RITT) within the LysRS that prevents the expression of the downstream gene. RITT are strong secondary RNA structures that destabilize the binding of the RNA-polymerase to target DNA and thus stop transcription of downstream genes. In the absence of lysine, an anti-terminator structure is formed within the LysRS, which then allows for transcription of the lysine transporter gene. Interestingly, Toledo-Arana *et al.* discovered that LysRS regulates expression of the upstream protein coding gene *lmo0799*, thus, functions as a 3′UTR regulator as well [8]. The transcript of *lmo0799* lacks an intrinsic 3′end terminator structure other than the LysRS that forms the RITT in lysine rich conditions. In contrast, lysine depletion leads to generation of a long transcript consisting of *lmo0799*-LysRS-*lmo0798* [9]. Additional regulator elements with a dual 5′ and 3′UTR function have been identified in *L. monocytogenes*, including the cobalamin, T-box, M-box and S-adenosylmethionine binding (SAM) riboswitches [9]. Furthermore, a recent study discovered that two SAM riboswitches can also function as *trans*-acting sRNA [24]. SAM-binding leads to formation of a terminator structure, also known as SAM riboswitch element A (SreA), which blocks expression of downstream genes *lmo2419*, *lmo2418* and *lmo2417* that are involved in the cysteine or methionine metabolism. Strikingly, SreA also diffuses and binds to the 5′UTR of the *prfA* mRNA preventing its translation. Because the 5′UTR thermosensor of *prfA* exhibits a closed stem-loop structure at low temperatures, thus hiding the SD site, binding of SreA to *prfA* predominantly occurred at 37 °C, when the *prfA* thermosensor exhibits its open structure and allows SreA to target the SD region. These findings outline the complexity of the prokaryotic regulation by non-coding RNAs that are rather multifunctional and emphasize the potential flexibility of how bacteria control the expression of essential virulence genes.

Furthermore, a regulatory role in the connection between nutrient availability and growth was recently shown for the flavin mononucleotide (FMN) riboswitch [37]. Addition of roseoflavin, a riboflavin analog, which is the natural ligand of the FMN riboswitch, inhibited the expression of the downstream riboflavin transporter gene, but also drastically limited growth of *Listeria.* In contrast, mutation of the FMN riboswitch allowed the pathogen to proliferate in the presence of roseoflavin. In conclusion, this study provides evidence that the FMN riboswitch directly controls bacterial growth.

## 5. *Trans-*Acting sRNAs

*Trans*-acting small non-coding RNAs (sRNAs) are encoded in intergenic regions (IGR) distal to the target gene. To date, 101 sRNAs (including SreA) have been identified in *L. monocyotenes*, predominantly by using whole genome tiling-arrays and RNA-Seq [8–10]. However, this number may be underestimated since it was discovered that riboswitches can also act as *trans*-acting sRNAs [24]. The entire set of 21 sRNAs previously discovered by other methods was confirmed by whole genome tiling arrays [9]. With a few exceptions, sRNAs usually measure less than 500 nucleotides in length, while most are ranging between 50–150 nucleotides.

Although functional data on sRNAs is limited, recently some sRNAs, including *rliB*, *rli31*, *rli33-1*, *rli38* and *rli50* were shown to have a role in virulence and growth of *L. monocytogenes* [9,10]. Except for *rli31,* these sRNAs are absent in *L. innocua* and other apathogenic strains of *Listeria*. A *rli38* knockout strain displayed attenuated growth in several mouse organs [9]. Δ*rli31,* Δ*rli33-1* and Δrli50* (due to an overlap between *rli50* and *rli112* in the intergenic region, the authors decided to delete *rli50* and the distal part of *rli112* to create Δrli50*) had decreased survival rates in infected murine macrophages, mouse organs and in an invertebrate infection model, when compared to the wild type strain, indicating an important role in virulence [10]. Δ*rliB* displayed affected virulence, although it colonized the liver faster than the wild type strain [9].

29 sRNAs were shown to be exclusively transcribed by bacteria within infected host cells, indicating a role in response to intracellular stress [10]. Interestingly, in *Listeriae* grown in blood, five of these sRNAs (LhrC1-5*)* displayed expression patterns similar to that of the virulence gene locus and virulence genes *vip* (*lmo0320*), *uhpT* and *inlC,* suggesting a possible role in virulence regulation [9].

The complexity of regulatory networks by sRNAs is increased by potential interactions between sRNAs [9]. Considering, that translation of a certain mRNA may only occur when mRNA levels prevail over levels of its regulatory RNA, sRNA/sRNA interactions could partially neutralize destabilizing effects of regulatory RNAs and favour mRNA translation. Reliable target prediction of sRNAs and experimental confirmation is therefore essential and currently an intensively investigated field.

## 6. Anti-Sense RNAs

Anti-sense RNAs (asRNAs) are encoded on the complementary DNA strand to the target transcript. Among asRNAs, different types and mechanisms of action can be distinguished depending on the localization on the opposite strand: (i) asRNAs that cover one or more ORFs without overlap to the flanking regions and (ii) 5′ or 3′UTRs of an mRNA overlapping with the mRNA encoded on the opposite strand.

In the first case, promoter as well as terminator of the asRNA is located in a region between the ORFs located at the opposite strand. The length of asRNA substantially varies and may range from a few bases to kilobases. Certain asRNAs may target only the SD region of a transcript while others cover several ORFs.

Overlapping 5′UTRs occur when the promoter of a certain gene is located within the targeted opposite divergent ORFs, but transcription of this gene ends upstream of the target ORF promoter. Overlapping 3′UTRs are produced when the promoter of the asRNA lies downstream of the target ORF terminator region and the transcription of the asRNA terminates within the ORF of the opposite strand.

More than 50 anti-sense regulatory RNAs and overlapping UTRs have been discovered in *L. monocytogenes* [9,10]. Although the majority of asRNAs identified so far are less than 150 nucleotides in size and represent bona fide asRNAs, several large UTRs have also been discovered. Functional and mechanistic data on the role of asRNAs is very limited. 3 asRNAs were confirmed by northern blots [9]. Interestingly, these asRNAs cover more than one ORF. For instance, the promoter of asRNA *anti2095* lies on the opposite strand of *lmo2095* and covers, both *lmo2096* and *lmo2097*, which encode for hypothetical proteins that may be implicated in carbohydrate metabolism. A similar organization was observed for *anti2325 and anti2394* [9].

Furthermore, the genome of *L. monocytogenes* harbours 13 overlapping 3′UTRs (*anti0360*, *anti0945*, *anti0946*, *anti2260* [10] and *anti0111*, *anti0734*, *anti1909*, *anti1910*, *anti1980*, *anti1981*, *anti2044*, *anti2045* and *anti2259* [9]) as well as 7 overlapping 5′UTRs (*anti0943*, *anti0977*, *anti2224* [10] and *anti0306*, *anti0647*, *anti0648* and *mogR* [9]). So far, only *mogR* has been characterized in further detail. MogR is a transcriptional repressor of flagellum genes *lmo0675, lmo0676 and lmo0677. mogR* possesses two promoters, P1, which is localized 1697 nucleotides from the start codon and P2 at 45 nucleotides from the ATG. Transcription start at P1 generates a long overlapping 5′UTR that covers three genes required for flagellum synthesis in *L. monocytogenes*, thus decreasing pathogen motility. In line with this, overexpression of the long *mogR* transcript was shown to reduce the motility of *L. monocytogenes*. Interestingly, P1 also contains a σ^B^ box. A subsequent experiment with a σ^B^ isogenic deletion mutant showed that expression of the long anti-sense transcript starting at P1 was directly regulated by σ^B^. Consequently, σ^B^ dependent *mogR* induction at P1 resulted in the generation of a long *mogR* transcript resulting in decreased motility of *L. monocytogenes* [9]. This mechanism emphasizes a possible role of regulatory RNAs in the ability to colonize infected host organs.

## 7. Role of Hfq and Transcriptional Control of Regulatory RNAs

Interaction of *trans*-acting sRNAs with target mRNA often requires the RNA chaperone Hfq [23]. This protein increases the stability of sRNA/mRNA hybridizations by an unknown mechanism. In contrast to other Gram-positive bacteria Hfq has an important role in virulence of *L. monocytogenes*, whereas Hfq is commonly indispensable for virulence in Gram-negative pathogens. However, to date, homologues of Hfq have been found in only half of the sequenced bacteria. Although Hfq was shown to be required for target interaction of several sRNAs, such as LhrA-C, others interact with the target mRNA independently from Hfq [38]. Possibly, further RNA binding proteins are involved in binding stabilization of sRNAs as suggested for other intracellular bacteria [39].

Regulatory RNAs undergo transcriptional deregulation in a variety of conditions, most prominently in bacteria grown in blood and bacteria located in the intestinal lumen or within macrophages [9,10]. Transcriptional regulation of several sRNAs was shown to be mediated by σ^B^, while PrfA and Hfq had little or no effect on sRNA expression [8,9].

## 8. Concluding Remarks and Future Outlook

Identification of a number of non-coding regulatory RNAs in many prokaryotic genomes has widened our view on the complexity of transcription regulation in bacteria. Recent developments in high-throughput techniques have been instrumental in this process. Tiling arrays and RNA-Seq have not only led to the detection of the vast majority of today’s known regulatory non-coding RNAs, but also channelled the discovery of new genes, the construction of the first operon map, the definition of untranslated regions and finally, the correction of previously annotated genes.

Although some insights have been obtained about how regulatory RNAs control mRNAs, precise functions and mechanisms remain largely unclear and need to be elucidated in further detail. Considering that only two years ago, the first sRNA has been described to have a role in virulence in *Listeria*, it becomes obvious that knowledge about the function of hundreds of regulatory RNAs and their target transcripts will have a remarkable impact on our understanding about several bacterial phenomena. For this process, *L. monocytogenes* serves as an unmatched model pathogen. Finally, regulatory RNAs may be attractive therapeutical targets to aim for with novel antibiotic treatments, such as PNAs [9,40] or possibly serve as diagnostic parameters.

## References

[1] Graves LM, Helsel LO, Steigerwalt AG, Morey RE, Daneshvar MI, Roof SE, Orsi RH, Fortes ED, Milillo SR, den Bakker HC (2010). *Listeria marthii* sp. nov., isolated from the natural environment, Finger Lakes National Forest. Int. J. Syst. Evol. Microbiol.

[2] Hain T, Chatterjee SS, Ghai R, Kuenne CT, Billion A, Steinweg C, Domann E, Karst U, Jansch L, Wehland J (2007). Pathogenomics of *Listeria* spp. Int. J. Med. Microbiol.

[3] Vazquez-Boland JA, Kuhn M, Berche P, Chakraborty T, Dominguez-Bernal G, Goebel W, Gonzalez-Zorn B, Wehland J, Kreft J (2001). *Listeria* pathogenesis and molecular virulence determinants. Clin. Microbiol. Rev.

[4] Leclercq A, Clermont D, Bizet C, Grimont PA, Le Fleche-Mateos A, Roche SM, Buchrieser C, Cadet-Daniel V, Le MA, Lecuit M, Allerberger F (2010). *Listeria rocourtiae* sp. nov. Int. J. Syst. Evol. Microbiol.

[5] Hof H, Szabo K, Becker B (2007). Epidemiology of listeriosis in Germany: a changing but ignored pattern. Dtsch. Med. Wochenschr.

[6] Chico-Calero I, Suarez M, Gonzalez-Zorn B, Scortti M, Slaghuis J, Goebel W, Vazquez-Boland JA (2002). Hpt, a bacterial homolog of the microsomal glucose- 6-phosphate translocase, mediates rapid intracellular proliferation in *Listeria*. Proc. Natl. Acad. Sci. USA.

[7] Cossart P, Toledo-Arana A (2008). *Listeria monocytogenes*, a unique model in infection biology: An overview. Microbes Infect.

[8] Oliver HF, Orsi RH, Ponnala L, Keich U, Wang W, Sun Q, Cartinhour SW, Filiatrault MJ, Wiedmann M, Boor KJ (2009). Deep RNA sequencing of *L. monocytogenes* reveals overlapping and extensive stationary phase and sigma B-dependent transcriptomes, including multiple highly transcribed noncoding RNAs. BMC Genomics.

[9] Toledo-Arana A, Dussurget O, Nikitas G, Sesto N, Guet-Revillet H, Balestrino D, Loh E, Gripenland J, Tiensuu T, Vaitkevicius K (2009). The *Listeria* transcriptional landscape from saprophytism to virulence. Nature.

[10] Mraheil MA, Billion A, Mohamed W, Mukherjee K, Kuenne C, Pischimarov J, Krawitz C, Retey J, Hartsch T, Chakraborty T (2011). The intracellular sRNA transcriptome of *Listeria monocytogenes* during growth in macrophages. Nucleic Acids Res.

[11] Zhang A, Wassarman KM, Rosenow C, Tjaden BC, Storz G, Gottesman S (2003). Global analysis of small RNA and mRNA targets of Hfq. Mol. Microbiol.

[12] Vogel J, Bartels V, Tang TH, Churakov G, Slagter-Jager JG, Huttenhofer A, Wagner EG (2003). RNomics in Escherichia coli detects new sRNA species and indicates parallel transcriptional output in bacteria. Nucleic Acids Res.

[13] Sittka A, Lucchini S, Papenfort K, Sharma CM, Rolle K, Binnewies TT, Hinton JC, Vogel J (2008). Deep sequencing analysis of small noncoding RNA and mRNA targets of the global post-transcriptional regulator, Hfq. PLoS Genet.

[14] Sittka A, Sharma CM, Rolle K, Vogel J (2009). Deep sequencing of *Salmonella* RNA associated with heterologous Hfq proteins *in vivo* reveals small RNAs as a major target class and identifies RNA processing phenotypes. RNA Biol.

[15] Sonnleitner E, Sorger-Domenigg T, Madej MJ, Findeiss S, Hackermuller J, Huttenhofer A, Stadler PF, Blasi U, Moll I (2008). Detection of small RNAs in *Pseudomonas aeruginosa* by RNomics and structure-based bioinformatic tools. Microbiology.

[16] Kreikemeyer B, Klenk M, Podbielski A (2004). The intracellular status of *Streptococcus pyogenes*: role of extracellular matrix-binding proteins and their regulation. Int. J. Med. Microbiol.

[17] Mangold M, Siller M, Roppenser B, Vlaminckx BJ, Penfound TA, Klein R, Novak R, Novick RP, Charpentier E (2004). Synthesis of group A streptococcal virulence factors is controlled by a regulatory RNA molecule. Mol. Microbiol.

[18] Perez N, Trevino J, Liu Z, Ho SC, Babitzke P, Sumby P (2009). A genome-wide analysis of small regulatory RNAs in the human pathogen group A *Streptococcus*. PLoS One.

[19] Halfmann A, Kovacs M, Hakenbeck R, Bruckner R (2007). Identification of the genes directly controlled by the response regulator CiaR in *Streptococcus pneumoniae*: five out of 15 promoters drive expression of small non-coding RNAs. Mol. Microbiol.

[20] Pichon C, Felden B (2005). Small RNA genes expressed from *Staphylococcus aureus* genomic and pathogenicity islands with specific expression among pathogenic strains. Proc. Natl. Acad. Sci. USA.

[21] Sorek R, Cossart P (2010). Prokaryotic transcriptomics: a new view on regulation, physiology and pathogenicity. Nat. Rev. Genet.

[22] Morita T, Maki K, Aiba H (2005). RNase E-based ribonucleoprotein complexes: mechanical basis of mRNA destabilization mediated by bacterial noncoding RNAs. Genes Dev.

[23] Gripenland J, Netterling S, Loh E, Tiensuu T, Toledo-Arana A, Johansson J (2010). RNAs: regulators of bacterial virulence. Nat. Rev. Microbiol.

[24] Loh E, Dussurget O, Gripenland J, Vaitkevicius K, Tiensuu T, Mandin P, Repoila F, Buchrieser C, Cossart P, Johansson J (2009). A trans-acting riboswitch controls expression of the virulence regulator PrfA in *Listeria monocytogenes*. Cell.

[25] Chatterjee SS, Hossain H, Otten S, Kuenne C, Kuchmina K, Machata S, Domann E, Chakraborty T, Hain T (2006). Intracellular gene expression profile of *Listeria monocytogenes*. Infect. Immun.

[26] Joseph B, Przybilla K, Stuhler C, Schauer K, Slaghuis J, Fuchs TM, Goebel W (2006). Identification of *Listeria monocytogenes* genes contributing to intracellular replication by expression profiling and mutant screening. J. Bacteriol.

[27] Hain T, Hossain H, Chatterjee SS, Machata S, Volk U, Wagner S, Brors B, Haas S, Kuenne CT, Billion A (2008). Temporal transcriptomic analysis of the *Listeria monocytogenes* EGD-e sigma B regulon. BMC Microbiol.

[28] McGann P, Raengpradub S, Ivanek R, Wiedmann M, Boor KJ (2008). Differential regulation of *Listeria monocytogenes* internalin and internalin-like genes by sigma B and PrfA as revealed by subgenomic microarray analyses. Foodborne. Pathog. Dis.

[29] Wang Z, Gerstein M, Snyder M (2009). RNA-Seq: A revolutionary tool for transcriptomics. Nat. Rev. Genet.

[30] Wurtzel O, Sapra R, Chen F, Zhu Y, Simmons BA, Sorek R (2010). A single-base resolution map of an archaeal transcriptome. Genome Res.

[31] Barry T, Kelly M, Glynn B, Peden J (1999). Molecular cloning and phylogenetic analysis of the small cytoplasmic RNA from *Listeria monocytogenes*. FEMS Microbiol. Lett.

[32] Nielsen JS, Olsen AS, Bonde M, Valentin-Hansen P, Kallipolitis BH (2008). Identification of a sigma B-dependent small noncoding RNA in *Listeria monocytogenes*. J. Bacteriol.

[33] Mandin P, Repoila F, Vergassola M, Geissmann T, Cossart P (2007). Identification of new noncoding RNAs in *Listeria monocytogenes* and prediction of mRNA targets. Nucleic Acids Res.

[34] Christiansen JK, Nielsen JS, Ebersbach T, Valentin-Hansen P, Sogaard-Andersen L, Kallipolitis BH (2006). Identification of small Hfq-binding RNAs in *Listeria monocytogenes*. RNA.

[35] Livny J, Teonadi H, Livny M, Waldor MK (2008). High-throughput, kingdom-wide prediction and annotation of bacterial non-coding RNAs. PLoS One.

[36] Johansson J, Mandin P, Renzoni A, Chiaruttini C, Springer M, Cossart P (2002). An RNA thermosensor controls expression of virulence genes in *Listeria monocytogenes*. Cell.

[37] Mansjo M, Johansson J (2011). The Riboflavin analog Roseoflavin targets an FMN-riboswitch and blocks *Listeria monocytogenes* growth, but also stimulates virulence gene-expression and infection. RNA Biol.

[38] Christiansen JK, Larsen MH, Ingmer H, Sogaard-Andersen L, Kallipolitis BH (2004). The RNA-binding protein Hfq of *Listeria monocytogenes*: role in stress tolerance and virulence. J. Bacteriol.

[39] Vogel J (2009). A rough guide to the non-coding RNA world of *Salmonella*. Mol. Microbiol.

[40] Mraheil MA, Billion A, Kuenne C, Pischimarov J, Kreikemeyer B, Engelmann S, Hartke A, Giard JC, Rupnik M, Vorwerk S (2010). Comparative genome-wide analysis of small RNAs of major Gram-positive pathogens: from identification to application. Microb. Biotechnol.

